# Improving equity in health care financing in China during the progression towards Universal Health Coverage

**DOI:** 10.1186/s12913-017-2798-7

**Published:** 2017-12-29

**Authors:** Mingsheng Chen, Andrew J. Palmer, Lei Si

**Affiliations:** 1 School of Health Policy & Management, Nanjing Medical University, 101Longmian Avenue, Jiangning District, Nanjing, 211166 People’s Republic of China; 2 Institute of Healthy Jiangsu Construction & Development, Nanjing, 211166 China; 3 Menzies Institute for Medical Research, University of Tasmania, Medical Science 1 Building, 17 Liverpool St (Private Bag 23), Hobart, TAS 7000 Australia

**Keywords:** Financing equity, Progressivity, Kakwani index, Universal Health Coverage

## Abstract

**Background:**

China is reforming the way it finances health care as it moves towards Universal Health Coverage (UHC) after the failure of market-oriented mechanisms for health care. Improving financing equity is a major policy goal of health care system during the progression towards universal coverage.

**Methods:**

We used progressivity analysis and dominance test to evaluate the financing channels of general taxation, pubic health insurance, and out-of-pocket (OOP) payments. In 2012 a survey of 8854 individuals in 3008 households recorded the socioeconomic and demographic status, and health care payments of those households.

**Results:**

The overall Kakwani index (KI) of China’s health care financing system is 0.0444. For general tax KI was −0.0241 (95% confidence interval (CI): −0.0315 to −0.0166). The indices for public health schemes (Urban Employee Basic Medical Insurance, Urban Resident’s Basic Medical Insurance, New Rural Cooperative Medical Scheme) were respectively 0.1301 (95% CI: 0.1008 to 0.1594), −0.1737 (95% CI: –0.2166 to −0.1308), and −0.5598 (95% CI: –0.5830 to −0.5365); and for OOP payments KI was 0.0896 (95%CI: 0.0345 to 0.1447). OOP payments are still the dominant part of China’s health care finance system.

**Conclusion:**

China’s health care financing system is not really equitable. Reducing the proportion of indirect taxes would considerably improve health care financing equity. The flat-rate contribution mechanism is not recommended for use in public health insurance schemes, and more attention should be given to optimizing benefit packages during China’s progression towards UHC.

## Background

Various countries have designed and implemented health sector reforms to bring about Universal Health Coverage (UHC), and the World Health Organization (WHO) has called for health systems to move towards UHC, where there are ‘key promotive, preventive, curative and rehabilitative health interventions for all at an affordable cost, thereby achieving equity in access’ [1]. The *2010 World Health Report* was devoted to UHC and it argued that financing systems need to be specifically designed to provide all people with access to the health services that they need, and to ensure that the use of these services does not expose the user to financial hardship, especially regarding poor and vulnerable groups [2, 3]. Consequently, policymakers must ensure that coverage is equitable and they must establish reliable sources of finance to fund health care. Improving the equity of health care financing has become a major policy goal in the development of UHC. However, policymakers often encounter challenges: does health care financing become equitable during the progression towards UHC?

China is reforming the way it finances its health care system as it moves towards UHC after the failure of the market-oriented approach to health care. China’s health care financing system has been influenced by economic transitions since the early 1980s, and the system was gradually reformed as it transitioned from a planned economic model to a market-oriented model [4]. Government health care spending declined as health care financing was decentralized. As a result, the share of public funding in the health care system decreased, while the proportion of private financing increased [5].

During the period of the planned economy, China’s social health insurance consisted of the Government Welfare Insurance Scheme (GWIS), the Labor Insurance Scheme (LIS) for those in urban areas and the Cooperative Medical Scheme (CMS) for those in rural areas. GWIS mainly covered civil servants, other government employees, veterans and college students, whereas LIS was for workers and their dependents across all the formal sectors of the economy [6].CMS played a key role in guaranteeing access to basic health services for the vast majority of the rural population, especially the poor [7]. Almost all of health care expenditures were funded by the government during the planned economy period. Taking the year 1980 as an example, OOP expenditure accounted for only 21.19% of all health care financing [8].

However, these health insurance schemes faced challenges brought about by the market-oriented economic reforms, which led to substantial changes in hospital management procedures and financing patterns. These reforms, coupled with the adoption of advanced medical technologies and economic inflation, became a major factor that increased health care costs. Along with the greater demands by employees for quality care, and the corresponding financial pressures, financing from GWIS and LIS greatly shrank and citizens had to pay much higher OOP expenditures for health care during the period of the market-oriented economy.

In 2000, OOP payments accounted for 58.98% of all health care financing [8]. The *2000 World Health Report* also noted that China had a very high per capita health care expenditure and an inequitable health care system [9]. The heavy dependence on OOP payments resulted in a segmented and tiered health care financing system, in which poor and vulnerable groups faced financial difficulties when accessing health care. The WHO indicated that not only do OOP payments cause financial stress and deter people from using health services, but they also cause inequity in health care financing [9]. The results of China’s 2003 national health services survey show that 48.9% of individuals who should have received outpatient care did not visit a health clinic. Among those who were admitted but did not use inpatient services, 75.4% could not afford the hospital charges [10].

The increase in costs and inequality in health care usage was considered to be a major crisis [11], and the Chinese government took steps to address these issues by establishing new types of health insurance schemes, as shown in Table 1. In 1998, the Chinese government established the Urban Employee Basic Medical Insurance (UEBMI), which covers urban workers in the formal sector. During the period 1998–2009, UEBMI gradually expanded to cover all urban workers in all types of organizations, including government institutions, state-owned and collective enterprises, private enterprises, enterprises with foreign investment, social organizations and private non-enterprise organizations. Moreover, the Urban Resident Basic Medical Insurance (URBMI) was established in 2007 for urban residents such as pre-school children, students, the disabled, the unemployed and elderly people without pensions [12]. In addition, the New Rural Cooperative Medical Scheme (NRCMS) was piloted in 2003 and officially implemented in 2007 to provide cover for rural residents.

**Table 1:** Summary of China’s current health insurance schemes

	UEBMI	NRCMS	URBMI
Starting year	1998	2003	2007
Target population	Urban workers	Rural farmers	Pre-school children, students, the disabled, the unemployed and elderly people without pensions
Financial contribution			
Government subsidy per person	0	240 yuan in 2012	120–230 yuan in 2012
Employer contribution	8% of salary	0	0
Individual contribution	2% of salary	60–65 yuan in 2012	70–160 yuan in 2012

Data source: National Health and Family Planning Commission of the People’s Republic of China. China health undertakings statistical bulletin, 1999–2010; National Health and Family Planning Commission of the People’s Republic of China. China health statistical yearbook, 2004–2010; Ministry of Human Resources and Social Security of the People’s Republic of China. Labour and social security undertakings statistical bulletin, 1992–2010

Currently, China’s health care financing sources consist of general taxation, OOP payments and public health insurance schemes (UEBMI, URBMI and NRCMS). In 2009, the Chinese government announced that it was to establish UHC by extending the coverage of UEBMI, URBMI and NRCMS in order to provide safe, effective, convenient and affordable health services to all Chinese people by 2020 [13]. As a result of the Chinese government’s attempts to accelerate the establishment of UHC by expanding the coverage of the three public health insurance schemes, in 2012, UEBMI, URBMI and NRCMS covered 274 million, 296 million and 802 million individuals, respectively. In 2012, the proportions of the relevant population covered by UEBMI, URBMI and NRCMS were 95.1, 89.2 and 97.3%, respectively [14]. However, 4.4% of the population was still not covered by any type of health insurance scheme and these people had to pay OOP for health care [14].

The Chinese government’s initiatives have expanded health coverage and they have attempted to encourage progressive payments over regressive payments, with the overall aims of reducing OOP payments and improving the equity of health care financing. Contribution to health care finance has been considered a redistribution of the disposable income of households [15, 16]. Progressive payments refer to the rich contribute a greater proportion of health care payments than the poor in comparison with their ability to pay (ATP). In contrast, regressive payments refer to the poor contribute a greater proportion of health care payments than the rich in comparison with ATP. However, cross-subsidization from the rich to the sick poses a potential challenge to UHC. For example, the individual contributions associated with UEBMI were a fixed proportion of employees’ salaries, whilst the individual contributions associated with URBMI and NRCMS were flat-rate premiums, regardless of each individual’s ATP. Although solidarity with the poor is widely supported in many countries [17], progressivity of health care finance may affect people’s willingness to participate in a health insurance scheme.

Some researchers have questioned the claim that poor individuals who are covered by URBMI or NRCMS contribute a greater share of the health care payments to health insurance schemes than the rich. Accordingly, evaluating the distribution of health care financing has become fundamental to assessing China’s progression towards UHC. However, few empirical studies provide evidence on the actual degree of financing equity. It is intended that this study will help to clarify the positive and negative aspects of China’s health care financing system, and thereby discover flaws in the financing mechanisms, which are heavily influenced by UHC initiatives.

## Methods

### Data sources

The data for the analyses came from a household survey in 2013 in North Jiangsu Province, China, which recorded the information in 2012. In terms of per capita gross domestic product (GDP), North Jiangsu, in the center of East China, is middle-ranked in China.

Adopting a multistage stratified random sampling method, the survey randomly selected five counties or county-level cities, and then five townships or neighborhoods were selected from each of these. In turn, two communities were selected from each of the townships or neighborhoods. About 60 households from each of the communities were then randomly selected, giving a total of 3008 households with 8854 individuals, as shown in Table 2.

**Table 2:** Descriptive statistics and socioeconomic characteristics of the sampling data by income quintile

Income quintiles	No. of families	No. of individuals	Per capita household expenditure	OOP
1—poorest	602	1767	5486.63 (5335.91 to 5637.35)	617.23 (559.71 to 674.76)
2	602	1780	10,355.37 (10,261.41 to 10,449.34)	932.15 (825.89 to 1038.41)
3	601	1775	14,558.69 (14,456.95 to 14,660.44)	1143.78 (1021.26 to 1266.30)
4	602	1762	20,261.31 (20,096.49 to 20,426.14)	1808.89 (1616.48 to 2001.30)
5—richest	601	1770	38,641.08 (37,205.61 to 40,076.54)	5169.61 (4224.93 to 6114.29)
Total	3008	8854	17,854.81 (17,352.02 to 18,357.60)	1933.52 (1729.42 to 2137.62)

Data source: Author’s analysis from the sample of household survey

Note: All expenditures are presented in CNY. 95% confidence intervals are in the parentheses

The survey was administered via household interviews. Within each sampled household, all household members aged 15 years and older were interviewed. Information on the children aged under 15 years was obtained via their guardians, as was information on adults with incapacities who required guardians. The face-to-face interviews were carried out by trained data collectors who used a structured questionnaire. This questionnaire contained a series of questions regarding the socioeconomic and demographic characteristics of each household and its members, including expenditure, number of household members, gender, age, employment status, earnings and education status. With regard to household expenditure, monthly expenditures on food, water, transport, housing, clothing, electricity, communications, education, fuel, entertainment, tour, health care and other expenditures were recorded. These data covered the 12-month period prior to the interviews. Household expenditure was recorded by the head of the household, or by members of the household who were familiar with the household’s affairs. Data on health care expenditure were collected using the interviewees’ medical records. The survey was confidential and personal identifiers were not collected. The study was approved by the Academic Research Ethics Committee of Nanjing Medical University.

Health care payments were computed using three data sources: the survey described above, the tariffs for tax and the contribution rates associated with UEBMI. The tariffs for general taxation were collected from the *China Price Statistical Yearbook* [14], while the contribution rates associated with UEBMI were obtained from the *Jiangsu Statistical Yearbook* [18].

General taxation is an important source of funding for health care in China. A variety of tax revenues exist, including excise taxes on food, drink, accommodation, alcohol, cigarettes, entertainment, gas and electricity, and various other consumption taxes. The taxes were estimated by applying the specific tax rates to the corresponding data on expenditures collected in the survey.

With regard to UEBMI, the household financing contributions were estimated by multiplying the contribution rate associated with UEBMI by the salaries of the relevant workers. With regard to URBMI, the annual premium was a flat-rate contribution. Each household was required to pay the same premium, due to the difficulty faced by the insurance agencies associated in identifying the socioeconomic status of each household. The flat amounts were obtained directly during the household interviews and they were aggregated at the household level. The same method was used for estimating the financing contribution associated with NRCMS.

Data on OOP payments during the two weeks prior to each interview were obtained during the survey, directly from the interviewees.

### Data analysis

The unit in the analysis of financing progressivity was the household. Expenditures and health care payments were aggregated at the household level. The household expenditure was used as the measurement of ATP [19]. The household expenditure was adjusted for household size and composition in order to obtain adult equivalent estimates. The number of adult equivalent household members was defined as follows:

$$ AE={\left(A+\alpha K\right)}^{\beta } $$

where *A* is the number of adults in the household, *α* is the cost of children, *K* is the number of children and *β* is the degree of economies of scale [19]. The values of *α* and *β* were assumed to be 0.5 and 0.75, respectively [20]. The population was ranked by ATP and grouped into quintiles. Household health care payments were also adjusted for household size and composition in order to obtain adult equivalent estimates.

The most direct means of assessing the progressivity of health care payments is to examine how the cumulative proportion of health care payments changes with the cumulative proportion of the population, ranked by ATP. Specifically, a progressivity analysis measures departures from proportionality in relation to health care payments and ATP.

The equity of health care financing is measured using the Kakwani index (KI), which is calculated as follows:

π_K_ = C – G

where C is the concentration index (CI) for health care payments and G is the Gini coefficient associated with the ATP variable [21]. The CI is a measure for assessing the proportionality of health care payments within a defined population and it is not a standard approach for the assessment of the equity of health care financing. The KI was used to estimate the degree of equity in the health care financing system. The π_K_ value ranges between −2 to 1, with a positive number indicating progressivity, and a negative number indicating regressivity. A π_K_ value of 0 indicates proportionality [19]. Progressivity (regressivity) indicates that the rich (poor) contribute a larger proportion of health care payments than the poor (rich) in comparison with ATP [19].

Computing the CI and the Gini coefficient requires directly relating the covariance between variables and the households’ fractional ranks according to their ATP [22, 23]. The estimates of the CI and the Gini coefficient can be obtained from ordinary least squares (OLS) regression of the health care payment variables and ATP, respectively, on the households’ fractional rank according to the ATP distribution [19, 24], as follows:

$$ 2{\upsigma}^2\left(\frac{{\mathrm{Y}}_{\mathrm{i}}}{\upvarphi}\right)=\alpha +\beta {\mathrm{X}}_{\mathrm{i}}+\varepsilon $$

where Y_i_ is the health care payment or ATP of household i, φ is the mean health care payment or ATP, X_i_X_i_ is the household fractional rank according to the ATP distribution and σ^2^ is its variance. The OLS value of β is an estimate of the CI or the Gini coefficient, depending on the variables used in the regression.

As the KI is the difference between the CI and the Gini coefficient, both of which can be computed using the regression method described above, its value can be computed using a regression of the following form [19]:

$$ 2{\sigma}^2\left[\frac{s_i}{\mu }-\frac{t_i}{\eta}\right]=\alpha +\theta {X}_i+\varepsilon $$

where *s*_*i*_ is the health care payment of household *i*, *μ* is an estimate of its mean, *t*_*i*_ is the ATP variable, *η* is an estimate of its mean, *X*_*i*_ is the household fractional rank according to the ATP distribution and *σ* ^*2*^ is its variance. The OLS value of *θ* is an estimate of the KI. The overall KI of the health care financing system can be computed by taking the weighted sum of the individual KIs for each source of finance, where the weights are equal to the proportions of revenue collected from each source.

In addition, dominance test was used after the progressivity analysis. In order to determine whether the health care financing mechanisms reduce inequity, in the sense that poor individuals contribute a smaller proportion of their wealth to the health care financing system than wealthy individuals, tests were conducted to determine whether one concentration curve dominates (i.e., lies above) the Lorenz curve or another concentration curve. For dominance testing, the standard errors and differences between ordinates were computed to allow for between-curve dependence, where appropriate [25]. A multiple comparison approach to testing was adopted [26], with the null hypothesis defined as the curves being indistinguishable. This was tested against both dominance and the crossing of curves [27]. The null hypothesis was rejected in favor of dominance if there was at least one statistically significant difference between the ordinates of the two curves in one direction and no significant differences in the other direction across 19 evenly-spaced quintile points from 0.05 to 0.95. The null hypothesis was rejected in favor of crossing of curves if there was at least one statistically significant difference in each direction [28].

## Results

Table 3 presents the quintile-based income shares of per capita household expenditures and health care payments in North Jiangsu in 2012. The financing distribution, CIs, KIs, and dominance tests associated with each source of health care financing are also used to describe health care financing progressivity (Table 3).

**Table 3:** Distribution of household expenditure and the progressivity of the health care financing sources

Income quintiles	Per capita household expenditure	General taxation	UEBMI	URBMI	NRCMS	OOP	Overall
1—poorest	6.15%	6.37%	1.72%	11.35%	30.35%	6.39%	
2	11.61%	12.19%	7.30%	15.17%	24.96%	9.65%	
3	16.29%	17.13%	15.04%	21.88%	18.09%	11.82%	
4	22.71%	23.42%	25.01%	22.00%	13.58%	18.72%	
5—richest	43.24%	40.89%	50.93%	29.60%	13.02%	53.42%	
Gini/CI	0.3678 ** (0.3581 to 0.3772)	0.3436 ** (0.3342 to 0.3529)	0.4978 ** (0.4673 to 0.5283)	0.1940 ** (0.1515 to 0.2364)	−0.1921 ** (−0.2138 to −0.1704)	0.4572 ** (0.3985 to 0.5160)	
KI	\	−0.0241 ** (−0.0315 to −0.0166)	0.1301 ** (0.1008 to 0.1594)	−0.1737 ** (−0.2166 to −0.1308)	−0.5598 ** (−0.5830 to −0.5365)	0.0896 ** (0.0345 to 0.1447)	0.0444
Weight		0.3779	0.1576	0.0087	0.0097	0.4461	
Dominance test							
- Against the 45° line		D-	D-	D-	D+	D-	
- Against the Lorenz curve		D+	D-	D+	D+	D-	

Note: The 95% confidence intervals are in the parentheses; **implies statistical significance at the level of 0.01; *implies statistical significance at the level of 0.05; D+ (D-) implies that the concentration curve dominates (is dominated by) the line of equity or the Lorenz curve

The values of the CIs for all of the financing sources apart from NRCMS were statistically significantly positive. This confirms that the wealthy contribute a larger proportion of their ATP to the financing of health care than the poor, as is clear from the dominance tests against the 45° line (the line of equity). Out of all the positive CIs, the CI for UEBMI is the largest and the CI for URBMI is the smallest. With regard to NRCMS, the CI value is statistically significantly negative, implying that the wealthy contribute absolutely less to the financing of health care than do the poor through NRCMS.

The values of the KIs associated with UEBMI and OOP payments were statistically significantly positive. This indicates that the wealthy contribute a larger proportion of health care payments than the poor in comparison with ATP, as was clear from the dominance tests against the Lorenz curve. The KIs for general taxation, URBMI and NRCMS were statistically significantly negative, indicating that the wealthy contribute a smaller proportion of health care payments than the poor in comparison with ATP. Among these sources of health care finance, the KI for NRCMS was negative with a statistically significant magnitude, implying that the poor funded a much larger share of health care payments relative to ATP than the rich through NRCMS.

In summary, the financing associated with UEBMI and OOP payments was progressive, whereas the financing associated with general taxation, URBMI and NRCMS was regressive. The overall KI was 0.0444, indicating that it is a progressive health care financing system.

The relative progressivity of the different sources of finance was tested using dominance methods (Table 4). The results indicate that the concentration curve associated with UEBMI is dominated by all the others, and so it can be concluded that UEBMI is the most progressive source of finance, because the poor contributed the smallest proportion of UEBMI in all sources of finance compared with ATP. The next most progressive source of finance is OOP payments, the concentration curve for which is dominated by all the others except UEBMI. The concentration curve for general taxation is dominated by URBMI and NRCMS, whereas the curve for URBMI is dominated by NRCMS. Therefore, it can be concluded that NRCMS is the most regressive source of finance, since the poor contributed the largest proportion of NRCMS in all sources of finance compared with ATP. The rank in relation to progressivity of financing sources is consistent with the estimates of the KIs.

**Table 4:** Tests of dominance between concentration curves for different sources of health finance

	URBMI	General taxation	OOP	UEBMI
NRCMS	D	D	D	D
URBMI		D	D	D
General taxation			D	D
OOP				D

Note: D indicates that concentration curve of row source is dominated by (is more progressive than) that of column source

## Discussion

This study questioned whether there was evidence that China’s health care financing system was equitable during China’s progression towards UHC. Generally, it was not really equitable in the big picture. The sources of finance for China’s health care system primarily comprised general taxation, UEBMI, URBMI, NRCMS and OOP payments. The health care payments associated with UEBMI and OOP payments were both progressive, as the values of the associated KIs were statistically significantly positive. However, the health care payments associated with general taxation, URBMI and NRCMS were regressive, as the values of the associated KIs were statistically significantly negative. Using these KI values and the results of the dominance tests that compared different concentration curves, the relative progressivity (from highest to lowest) of the sources of finance for health care is as follows: UEBMI, OOP payments, general taxation, URBMI and NRCMS. Overall, the health care financing system was slightly progressive, since the overall KI was positive but close to zero.

With regard to general taxation, the tax burden demonstrated a pro-rich bias in the distribution of health care financing. This contradicted the findings in the literature that general taxation is a progressive mechanism to fund health care in both high- and middle-income countries [29]. In contrast to developed countries, where direct taxes comprise the majority of the general taxation system, indirect taxes dominate the general taxation system in China. This represents a pro-rich policy because the tax burden is transferred from the wealthier people to the lower-income echelons. Within the region of interest in the current study, indirect taxes (which, in China, include valued-added tax (VAT), excise tax and sales tax) accounted for 65.82% of general taxation in 2012 [18]. The high reliance on indirect taxes in China has resulted in the regressive effect of general taxation on China’s health care financing system. It is suggested that, in the move towards UHC, not only general tax collection should be increased through a variety of tax sources to fund the pool of UHC, but also general tax structure should be renovated by reduction on indirect taxes to improve financing equity of UHC.

With the goal of achieving UHC by 2020, efforts have been made to expand the coverage of existing health insurance schemes (UEBMI, URBMI, and NRCMS) to a wider Chinese population [30]. By 2012, over 95% of the total population has enrolled one of these three health insurance schemes [14]. Whilst China has made a big progress on the coverage, the equity and affordability of health care is still under investigated. The progressivity of public health insurance schemes differed between UEBMI, URBMI and NRCMS. UEBMI was the most progressive financing source, whereas URBMI and NRCMS were the second most regressive and the most regressive sources, respectively. These differences can be attributed to the individual contribution mechanisms used in the different schemes. UEBMI was jointly financed by employees and employers. The employees contributed around 2% of their salaries and the employers contributed around 7% of the employees’ salaries, although this proportion varied slightly depending on the region and the employee’s age [31]. This indicates that the UEBMI contribution was positively correlated with income. In contrast, there was a flat rate of individual contributions associated with both NRCMS and URBMI, as it was difficult for the insurance agencies to measure the income of rural households or urban households where household members often did not have stable jobs. Therefore, the insured population who enrolled in NRCMS and URBMI were required to pay the same premium, regardless of their ATP. This contribution mechanism explains why the KIs associated with these schemes were negative, which indicates an inequity that disadvantages the poorer members of society.

OOP payments were found to be progressive, indicating that the wealthier contributed a greater proportion of their ATP via direct payments than the poor. However, cautious interpretation is required when considering the KI and equity associated with the OOP payments, because the progressive distribution may be attributable to a ‘combined effect’ of the development of UHC in China. The ‘combined effect’ emerged after the initiation of China’s campaign to establish UHC (which involved extending financial risk protection and access to health services for the poor) as health care expenses were partly compensated for by insurance schemes. The OOP expenditure of the poor decreased and their share of OOP payments relative to their ATP tended to be smaller than that of the wealthier. On the other hand, unlike other sources of health care finance, OOP payments are post-payments, which means that care can only be provide to people who can finance their own health care. For example, in some middle- and low-income countries that are moving towards UHC, the wealthiest have no difficulty affording health care, even for highly-priced medical goods, at the cost of some co-payment. However, these co-payments represent obstacles to accessing health care for the poor and some middle-class people, because they cannot afford the expense [32, 33]. Consequently, these effects may explain the progressive distribution of OOP payments.

The relatively high level of OOP payments poses a challenge for China as it moves towards UHC. In the region of interest in this study, OOP payments accounted for 44.61% of the health care financing (Table 3). Heavy dependence on OOP payments encourages overuse by people who can pay and underuse by those who cannot. The high proportion of OOP payments in China can be attributed to the prioritization of China’s UHC plans, which focused on increasing the population coverage. It was reported that, in 2012, 95.4% of the population in North Jiangsu were covered by the various health insurance schemes. However, coverage of health services and costs was not as comprehensive. In the insurance list, the co-insurance rates associated with UEBMI, URBMI and NRCMS were 71.4, 57.2 and 46.6%, respectively [34]. If the required health care services were not on the insurance list, patients had to pay for them using OOP payments. Therefore, OOP payments remained at a high level during China’s progression towards UHC. In addition, this adversely affected patients’ care-seeking behavior, especially regarding the health-seeking behavior of the poor.

A limitation of our study is that the data were collected from a single province. The results might not represent the case for entire China and might not apply to the equity of health care finance in other provinces. This limitation notwithstanding, our study used relative scale of indices to evaluate the implementation of national policies and programs across the whole population. Accordingly, our study is less associated with specific regional economies or geography.

## Conclusion

The study shows that China’s health care financing distribution was slightly progressive during the progression to UHC. Financing via general taxation was regressive because indirect taxation dominates the general tax structure. Exemptions from indirect taxes, especially for the taxes that heavily impact on vulnerable groups, could play a large part in improving the equity of financing. The different financing contribution mechanisms of the public health insurance schemes resulted in different levels of progressivity. UEBMI was progressive, whereas URBMI and NRCMS were both regressive. Flat-rate contributions are not recommended and it is suggested that the contributions of the wealthy should be higher in order to cover the non-contributing members. Although OOP payments were progressive, this may be due to the underuse of health services by the poor. In addition, OOP payments still dominated China’s health care financing system. This indicates that the next phase of the development of UHC should focus on updating the benefit packages of the health insurance schemes, which could include extending the range of health services made available by the schemes and the proportion of the total costs of care to be covered by the insurance schemes.

## Abbreviations

ATP: Ability to pay
CI: Concentration index
CMS: Cooperative Medical Scheme
GWIS: Government Welfare Insurance Scheme
KI: Kakwani index
LIS: Labor Insurance Scheme
NRCMS: New Rural Cooperative Medical Scheme
OLS: Ordinary least squares
OOP: Out-of-pocket
UEBMI: Urban Employee Basic Medical Insurance
UHC: Universal Health Coverage
URBMI: Urban Resident Basic Medical Insurance
VAT: Valued-added tax
WHO: World Health Organization
